# When the ECG Misleads: Artifacts, Mimics, and the Art of Interpretation

**DOI:** 10.1016/j.jaccas.2026.108355

**Published:** 2026-05-20

**Authors:** Mervat Aboulmaaty

**Affiliations:** a Heart Beat Centre, Ain Shams University, Cairo, Egypt; b Cardiology Department, Ain Shams University, Cairo, Egypt

**Figure:**
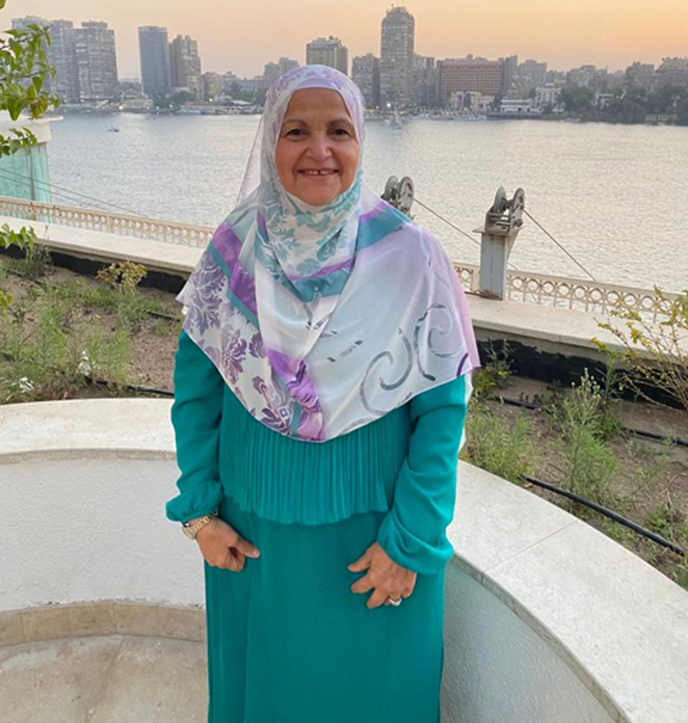

The electrocardiogram (ECG) is one of the most common and easy-to-get tests in cardiovascular medicine. It gives you an instant look at cardiac electrophysiology and is often the first and only objective record of a patient's cardiac health. Even though the ECG is everywhere and seems to be very accurate, it can still be misread. Patterns that seem to be diagnostic may not be, and small details may be missed.

A patient was referred following catheter ablation for presumed atrial fibrillation (AF), a diagnosis initially based on Holter monitoring. Upon careful re-evaluation of the original recording, the rhythm was found not to represent AF, but rather to represent an artifact due to signal interference. As a result, the patient had an invasive procedure for a condition that was not present.

This case demonstrates that artifacts can be misinterpreted as arrhythmia, reinforcing the need for careful evaluation of ECG signals before proceeding with invasive management.

The reliability of an ECG is primarily dependent on signal quality. The tracing can be distorted by motion, tremors, poor electrode contact, and electrical interference, which can make patterns that look like real arrhythmia or ischemia. International standards highlight the importance of placing electrodes correctly, and reducing noise and adjustment of filters in each setting are key steps for accurate interpretation.^1^ But in everyday practice, these steps are often not given enough attention.

ECG artifacts are well documented and can resemble many serious conditions, such as AF, ventricular tachycardia, and ST-segment elevation myocardial infarction.^2^^,^^3^ Cases of pseudo-ventricular tachycardia resulting from artifacts have resulted in unnecessary diagnostic and therapeutic interventions.^2^ Recognition of artifacts is essential for ensuring patient safety.

Acquiring an ECG for each patient is a very important task, as it may be the only ECG recorded in their lifetime and could reveal a serious underlying disease. It might uncover conditions such as cardiomyopathy, AF, long QT syndrome, or Brugada syndrome.

Obtaining a clear ECG signal is essential. This requires avoiding external interference, minimizing patient movement or tremors, and ensuring good electrode contact with the skin and limbs.

Using artificial intelligence tools to analyze ECGs and provide provisional diagnoses with measurements can be very helpful. However, it is important to remember that these tools are not a substitute for clinical judgment.

Always review the ECG carefully: assess the P-wave, QRS complex, QT interval, U-wave, and ST-segment. Do not rely solely on machine interpretation—human evaluation is critical, as artificial intelligence cannot replace clinical expertise in ECG diagnosis.

Be aware of ECG mimics—conditions such as electrolyte disturbances, early repolarization, or artifact can resemble serious pathology and lead to misdiagnosis. Always interpret findings in the clinical context.

Look for hidden clues within the ECG, as subtle changes may be the key to diagnosing life-threatening conditions that are not immediately obvious.

Finally, always double check the ECG quality and connections, and ensure that any sources of interference are minimized.

## Funding Support and Author Disclosures

The authors has reported that she has no relationships relevant to the contents of this paper to disclose.
